# Progressive scoliosis associated with microphthalmia with limb anomalies: A case report

**DOI:** 10.1097/MD.0000000000033414

**Published:** 2023-03-24

**Authors:** Yoshiro Yoshikawa, Chikashi Yamakawa, Takanao Shimabukuro, Hideo Kinjo, Shogo Fukase, Hiromichi Oshiro, Ryo Katsuki, Yasunori Tome, Kotaro Nishida

**Affiliations:** a Department of Orthopedic Surgery, Graduate School of Medicine, University of the Ryukyus, Okinawa, Japan.

**Keywords:** case report, microphthalmia with limb anomalies, posterior fusion, syndromic scoliosis, Waardenburg anophthalmia syndrome

## Abstract

Microphthalmia with limb anomalies is a rare, autosomal recessive, multiple congenital anomaly syndrome. Patients with this syndrome particularly present with monocular or bilateral anophthalmia/microphthalmia and distal limb anomalies. However, details regarding associated spinal deformities have not been fully elucidated.

**Patient concerns:**

A 12-year-old girl initially presented with progressive scoliosis, who was previously diagnosed with microphthalmia with limb anomalies. However, 4 years after the initial visit, the scoliosis deformity gradually progressed. The patient and family requested the surgical treatment to preserve standing/sitting balance.

**Diagnoses:**

She was diagnosed with microphthalmia with limb anomalies and progressive scoliosis.

**Interventions:**

A posterior corrective fusion surgery (including a pelvic fusion) was performed to prevent future standing/sitting imbalance.

**Outcomes:**

Significant improvement of spinal deformity was observed, with no adverse events.

**Lessons:**

This report demonstrated a case of progressive scoliosis associated with microphthalmia with limb anomalies. A posterior corrective spinal fusion was effective to preserve standing/sitting balance. To the best of our knowledge, this is the first report of surgical treatment of progressive scoliosis associated with microphthalmia with limb anomalies.

## 1. Introduction

Microphthalmia with limb anomalies is a rare, autosomal recessive, multiple congenital anomaly syndrome.^[1]^ Patients with this disease are characterized by monocular or bilateral anophthalmia/microphthalmia and distal limb anomalies. A few reports on spinal deformities in such patients have been published. ^[2,3]^ However, there are no previous reports on progressive scoliosis in patients with microphthalmia with limb anomalies. Herein, we present the case of one such patient, who was successfully treated by posterior corrective fusion surgery.

## 2. Case report

**Figure 1.**
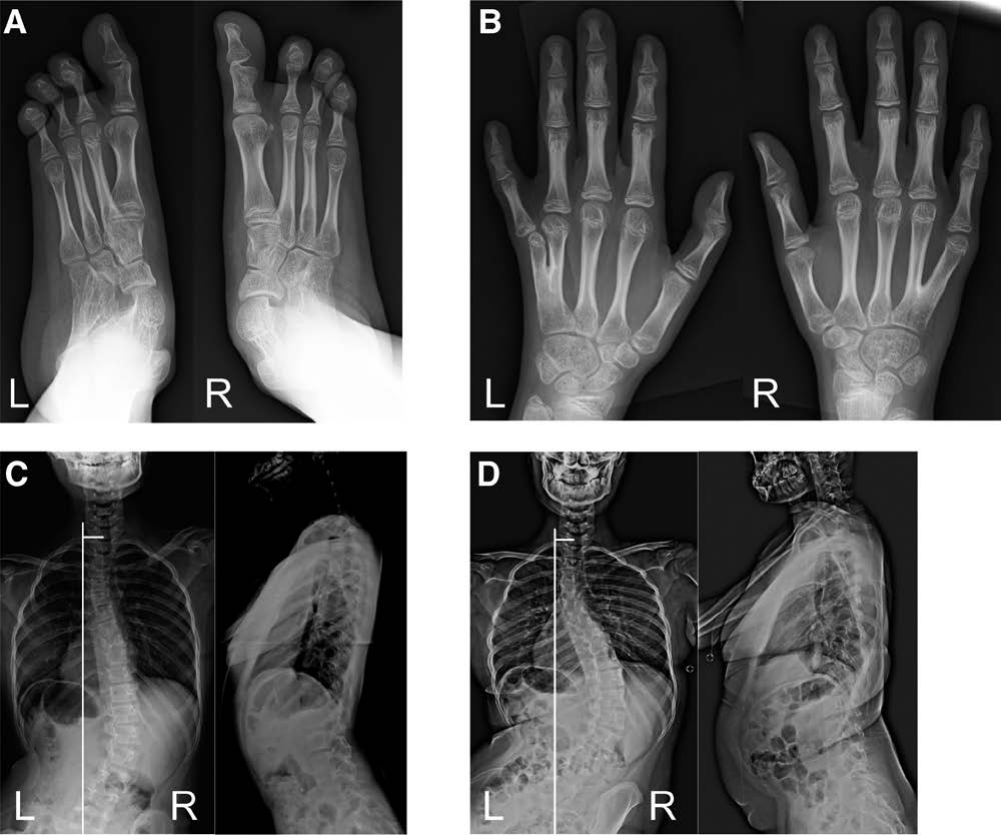
Progressive scoliosis due to microphthalmia with limb anomalies. (A) Four toes and second- and third-toe cutaneous syndactyly are observed bilaterally. (B) Fourth and fifth metacarpal synostoses are observed in both hands. (C) Initial radiographs. Left, the Th7-L4 Cobb angle is 47°. The C7-center sacral vertical line (CSVL) is shifted 26 mm to the right. Right, the sagittal vertical axis (SVA) is 21 mm. The angle of thoracic lordosis (TL) is 21°. The angle of pelvic obliquity (PO) is 10°. (D) The patient’s scoliosis worsened 4 years after the initial visit. Left, the Th7-L4 Cobb angle is 74°. The C7-CSVL is shifted 28 mm to the right. Right, the SVA is 45 mm. The angle of TL is worse at 29°. The angle of PO has progressed to 13°.

**Figure 2.**
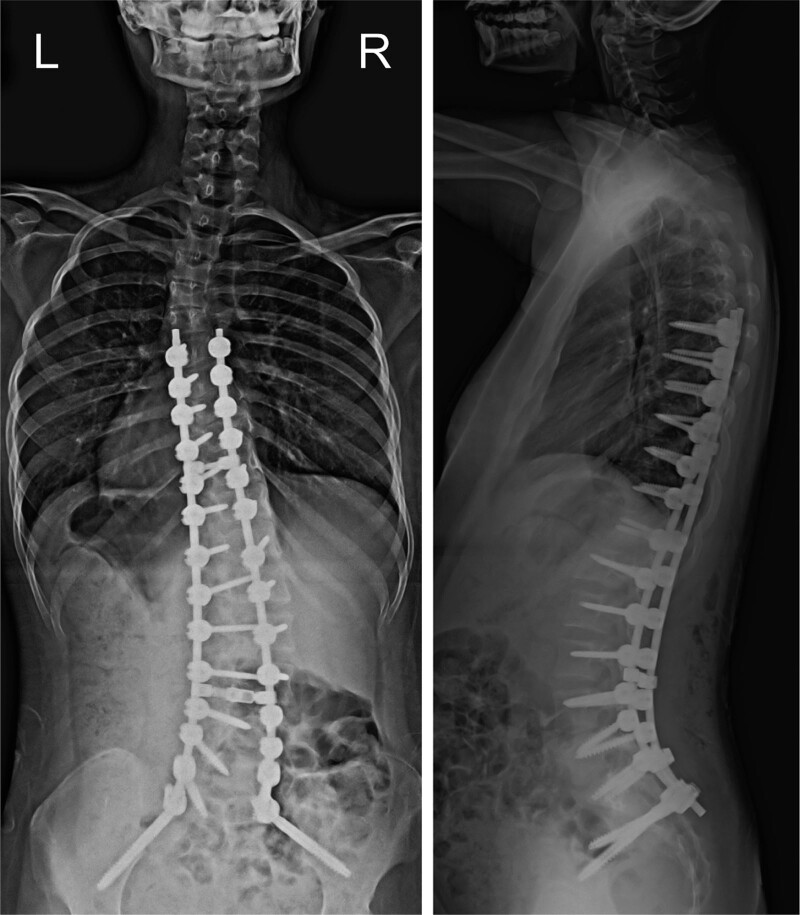
Radiographs taken after posterior corrective fusion of Th6 to the sacral alar-iliac was performed. Left, the Th7-L4 Cobb angle has improved to 28°. The C7-CSVL is shifted 2 mm to the left. Right, SVA is 2 mm. The angle of thoracic kyphosis is 14°. CSVL = center sacral vertical line, SVA = sagittal vertical axis.

**Figure 3.**
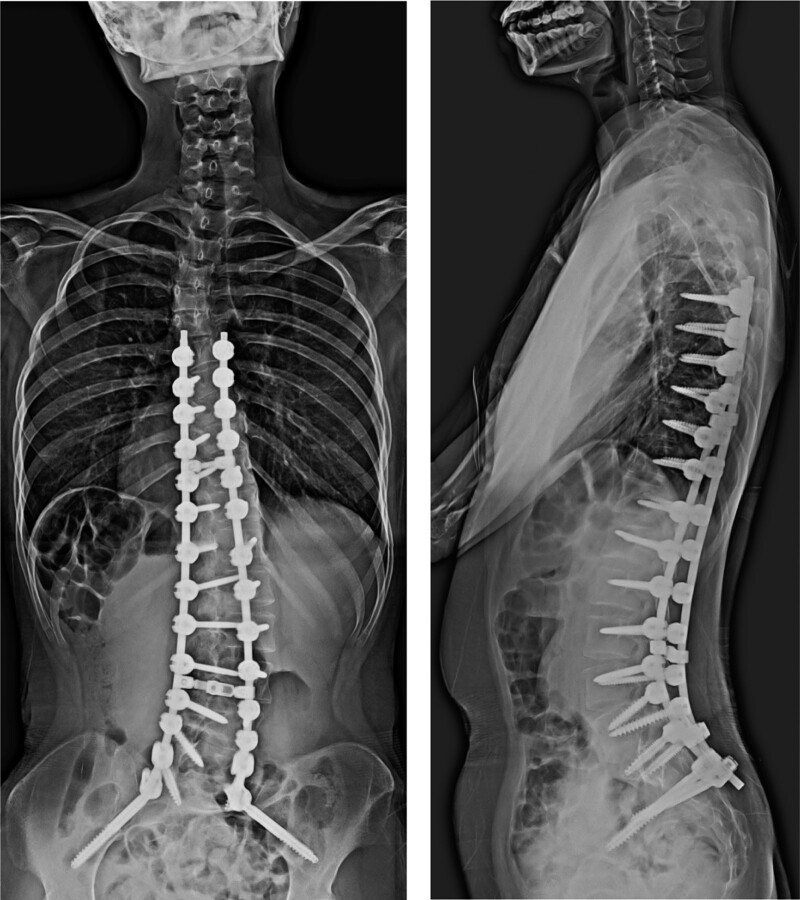
Radiographs taken a year and a half later. No progression of the scoliosis is observed. Left, the C7-CSVL is shifted 6 mm to the right. Right, the SVA is 7 mm. The angle of thoracic kyphosis is 13°. CSVL = center sacral vertical line, SVA = sagittal vertical axis.

A 12-year-old girl with microphthalmia and limb anomalies was referred to our department for progressive scoliosis. She had 4 toes on both feet (Fig. 1A), second- and third-toe cutaneous syndactyly, bilateral 4th and fifth metacarpal synostoses (Fig. 1B), bilateral fibular dysplasia, mental retardation, and epilepsy. Her older brother was also followed up for the same disease with mild scoliosis at another hospital. A primary curvature to the right, an elevated left shoulder, and a prominent right hip were observed on physical examination. She walked with her trunk leaning to the right side. No muscle weakness was observed. Both upper and lower limb tendon reflexes were normal. Hoffman, Troemner, and Babinski reflexes were negative. On radiography, the Th7-L4 Cobb angle was 47° in the standing position, C7-center sacral vertical line was shifted by 26 mm to the right, sagittal vertical axis was 21 mm, and thoracic lordosis (TL) angle was 21°. The angle of pelvic obliquity (PO) measured by Osebold technique ^[4]^ was 10° with standing radiograph (Fig. 1C). She refused orthotic therapy as the patient had mental retardation. and opted for outpatient management. Her scoliosis initially progressed gradually; however, 4 years later, it worsened rapidly. The Th7-L4 Cobb angle had progressed to 74°, C7- center sacral vertical line had shifted by 28 mm to the right (with marked pelvic rotation), sagittal vertical axis was 45 mm, TL angle was 29°, and PO angle was 13° (Fig. 1D). No physical and neurological symptoms were noted. The patient underwent surgery to avoid future respiratory disturbances and a coronal imbalance (which could affect the gait and sitting balance). Distal foundation with pelvic fixation was undertaken to reduce the coronal imbalance. A posterior corrective fusion of Th6 with the sacral alar-iliac was performed. After the surgery, the Cobb angle improved to 28°, TL was corrected, and the thoracic kyphosis angle was 14° (Fig. 2). No surgical complications, such as infections, instrument loosening/breakage, or correction loss, occurred after 18 months of follow up (Fig. 3).

## 3. Discussion

Microphthalmia with limb anomalies is also known as Waardenburg anophthalmia syndrome, ophthalmo-acromelic syndrome, and anophthalmia–syndactyly^[5]^; mutations in the secreted protein acidic and rich in cysteine-related modular calcium-binding protein 1 gene have been reported as its cause.^[3,6,7]^ Patients with this disease have monocular or bilateral anophthalmia/microphthalmia and distal limb anomalies. Additionally, delayed developmental milestones, internal organ abnormalities, and facial feature abnormalities have been reported in patients with this disease.^[8–10]^ A few cases of spinal deformities in patients with this condition, such as a cervical fusion with hemivertebrae^[2]^ and mild scoliosis,^[3]^ have been reported. Meanwhile, syndromic maicrophthalmia-1, the Lenz microphthalmia syndrome, has association with scoliosis.^[11]^ However, there are no previous reports on the surgical treatment of progressive scoliosis in patients with microphthalmia with limb anomalies.

This case demonstrates that posterior corrective fusion combined with pelvic fusion can correct progressive scoliosis associated with microphthalmia with limb anomalies. It should be pointed out that the range of spinal correction and fixation in ambulatory patients with syndromic scoliosis remains controversial.^[12]^ Previous studies have revealed that, especially in nonambulatory patients with neuromuscular scoliosis, extending instrumentation to the pelvis improves scoliosis correction and sitting balance if the angle of PO exceeds 10° to 15°.^[13–16]^ In this case, the patient was ambulatory at the time of surgery; however, the nature of her progressive scoliosis had caused the PO angle to increase significantly. Therefore, in this case, a posterior corrective fusion from Th6 to the sacral alar-iliac was performed to correct the Th7-L4 Cobb angle of 74° to deter further PO progression in the future. Favorable clinical outcomes, including Cobb angle correction and improvement in the gait/sitting balance, were noted at follow up.

The postoperative mid-term mortality rate for patients with severe scoliosis is low. However, the mortality beyond a 2-year follows up has rarely been reported.^[17]^ Although no correction loss or scoliosis progression was observed after 18 months of follow-up, the patient should continue with her follow up sessions.

## 4. Conclusion

We have described the first case of progressive scoliosis due to microphthalmia with limb anomalies; posterior spinal corrective fusion, including pelvic fixation, effectively corrected the progressive scoliosis, and coronal imbalance.

## Acknowledgments

We would like to thank Editage (www.editage.com) for English language editing.

## Author contributions

**Conceptualization:** Yoshiro Yoshikawa, Yasunori Tome.

**Data curation:** Yoshiro Yoshikawa, Chikashi Yamakawa, Takanao Shimabukuro, Hideo Kinjo, Shogo Fukase, Hiromichi Oshiro, Ryo Katsuki, Yasunori Tome.

**Formal analysis:** Yoshiro Yoshikawa.

**Investigation:** Yoshiro Yoshikawa, Chikashi Yamakawa, Yasunori Tome.

**Methodology:** Yoshiro Yoshikawa, Chikashi Yamakawa, Yasunori Tome, Kotaro Nishida.

**Project administration:** Yasunori Tome

**Resources:** Chikashi Yamakawa, Takanao Shimabukuro, Hideo Kinjo, Shogo Fukase.

**Supervision:** Yasunori Tome, Kotaro Nishida.

**Visualization:** Yoshiro Yoshikawa.

**Writing – original draft preparation:** Yoshiro Yoshikawa, Chikashi Yamakawa, Takanao Shimabukuro, Hideo Kinjo, Hiromichi Oshiro, Ryo Katsuki, Yasunori Tome.

**Writing – review and editing:** Yasunori Tome, Kotaro Nishida.
